# Attenuated glucose uptake promotes catabolic metabolism through activated AMPK signaling and impaired insulin signaling in zebrafish

**DOI:** 10.3389/fnut.2023.1187283

**Published:** 2023-05-26

**Authors:** Longwei Xi, Gang Zhai, Yulong Liu, Yulong Gong, Qisheng Lu, Zhimin Zhang, Haokun Liu, Junyan Jin, Xiaoming Zhu, Zhan Yin, Shouqi Xie, Dong Han

**Affiliations:** ^1^State Key Laboratory of Freshwater Ecology and Biotechnology, Institute of Hydrobiology, Chinese Academy of Sciences, Wuhan, China; ^2^College of Advanced Agricultural Sciences, University of Chinese Academy of Sciences, Beijing, China; ^3^Hubei Hongshan Laboratory, Huazhong Agriculture University, Wuhan, China; ^4^The Innovative Academy of Seed Design, Chinese Academy of Sciences, Wuhan, China

**Keywords:** *GLUT2*, homeostasis, insulin, lipolysis, proteolysis, zebrafish

## Abstract

Glucose metabolism in fish remains a controversial area of research as many fish species are traditionally considered glucose-intolerant. Although energy homeostasis remodeling has been observed in fish with inhibited fatty acid β-oxidation (FAO), the effects and mechanism of the remodeling caused by blocked glucose uptake remain poorly understood. In this study, we blocked glucose uptake by knocking out *glut2* in zebrafish. Intriguingly, the complete lethality, found in *Glut2*-null mice, was not observed in *glut*2^−/−^ zebrafish. Approxiamately 30% of *glut*2^−/−^ fish survived to adulthood and could reproduce. The maternal zygotic mutant *glut2* (MZ*glut2*) fish exhibited growth retardation, decreased blood and tissue glucose levels, and low locomotion activity. The decreased pancreatic β-cell numbers and *insulin* expression, as well as liver *insulin receptor a* (*insra*), fatty acid synthesis (*chrebp, srebf1, fasn, fads2*, and *scd*), triglyceride synthesis (*dgat1a*), and muscle mechanistic target of rapamycin kinase (*mtor*) of MZ*glut2* zebrafish, suggest impaired insulin-dependent anabolic metabolism. Upregulated expression of lipolysis (*atgl* and *lpl*) and FAO genes (*cpt1aa* and *cpt1ab*) in the liver and proteolysis genes (*bckdk, glud1b*, and *murf1a*) in muscle were observed in the MZ*glut2* zebrafish, as well as elevated levels of P-AMPK proteins in both the liver and muscle, indicating enhanced catabolic metabolism associated with AMPK signaling. In addition, decreased amino acids and elevated carnitines of the MZ*glut2* zebrafish supported the decreased protein and lipid content of the whole fish. In summary, we found that blocked glucose uptake impaired insulin signaling-mediated anabolism *via* β-cell loss, while AMPK signaling-mediated catabolism was enhanced. These findings reveal the mechanism of energy homeostasis remodeling caused by blocked glucose uptake, which may be a potential strategy for adapting to low glucose levels.

## Introduction

Fish are known to have a poor ability to use dietary carbohydrates. Overloading dietary carbohydrates may cause metabolic disorders in fish, such as fatty liver disease, stress response, and reduced growth performance (1, 2). Although the high carbohydrate diet challenge has been undertaken in several studies (3–6), the metabolic consequences of blocking glucose uptake under normal conditions are unknown. Insulin is well known to promote anabolic metabolism by stimulating lipid and protein synthesis (7), whereas adenosine 5′-monophosphate-activated protein kinase (AMPK) signaling promotes catabolic metabolism by switching on catabolic pathways but turning-off energy-consuming processes (8–11). Recently, studies in fish demonstrated that inhibited fatty acid β-oxidation by *cpt1b* and *pparab* deletion, or mildronate administration caused the remodeling of energy homeostasis by increasing glucose utilization and inhibiting amino acid breakdown through activating AMPK pathways (10, 12). However, the energy homeostasis remodeling of fish after blocking glucose uptake and the regulation of insulin and/or AMPK signaling remains unknown.

Glucose enters cells through facilitated diffusion regulated by a large family of glucose transporter proteins (GLUTs) (13), which contain 12 membrane-spanning helices with amino and carboxyl termini exposed to the cytosol. Glucose transporter 2 (GLUT2, also known as *SLC2A2*), is encoded by *solute carrier family 2 member 2*, which is expressed in various tissues, including the liver, intestine, kidney, pancreatic β-cell, and central nervous system (14–16). GLUT2 in mammals has been known to transport glucose in different tissues, such as the intestine, liver, and kidney (17). Inactivating GLUT2 mutations resulted in a condition associated with hepatomegaly, growth retardation, and Fanconi syndrome, which is characterized by glucose malabsorption, renal glucosuria, and transient neonatal diabetes (17, 18). Loss of GLUT2 in mice usually leads to death within 2–3 weeks of birth (19) and exhibits hyperglycemia and hypoinsulinemia, and elevated levels of glucagon and free fatty acids in plasma (20). Interestingly, transgenic re-expression of GLUT1 or GLUT2 in pancreatic β-cells rescues GLUT2-null mice from early death and restores glucose-stimulated insulin secretion (21, 22), which indicated that the function of GLUT2 in pancreatic β-cells plays an important role for survival. To date, the effect of *Glut2* on the normal maintenance of pancreatic β-cells and the survival of fish has not been reported.

The deletion of GLUT2 in the kidney improved glucose tolerance, reversed hyperglycemia, and normalized body weight in mice with diabetes and obesity (23). However, the deletion in the liver and kidney eliminated these improvements (23). It was revealed that GLUT2 participated in glucose absorption in the intestine, glucose-stimulated insulin secretion in β-cells, glucosensing capabilities, and food intake in the brain (13, 17). The differential physiological role of GLUT2 in systemic glucose homeostasis is tissue-specific, and this may be due to the differentiated systemic regulatory network of glucose in different tissues. On the other hand, given the crucial role of skeletal muscle in glucose metabolism, more attention should be paid to adaptive metabolic responses to glucose uptake attenuation. Studies in mice have shown that skeletal muscles are essential for regulating glucose metabolism as they are responsible for 70% of postprandial glucose uptake (24). The metabolic consequences of glucose uptake attenuation-mediated energy insufficiency would contribute to the elucidation of the systemic regulatory network of nutrient metabolism in non-mammalian vertebrates and provide an excellent model for dissecting the intrinsic association and regulatory network of insulin and AMPK signaling.

Zebrafish have been developed as an appropriate model for nutrient metabolism research (12, 25, 26). In this study, we constructed the *glut2*-deletion zebrafish model to block glucose uptake in fish and found that zebrafish with *glut2*-deletion did not completely die like mice, and surviving *glut2*-deletion zebrafish could reproduce. Therefore, these maternal zygotic mutant *glut2* (MZ*glut2*) zebrafish may be ideal models for studying overall nutrient metabolism after glucose loading. In addition, we found that *glut2*-deletion caused a decrease in the number of β-cells, which was related to the decrease of insulin-associated anabolic metabolism in surviving zebrafish. However, MZ*glut2* zebrafish may adapt to blocked glucose uptake by remodeling the metabolic pattern *in vivo* by reducing insulin-mediated anabolism and enhancing AMPK-mediated catabolism. These results suggest that *glut2* is important in regulating glucose uptake and is a key signal for maintaining energy expenditure in fish. Thus, we reveal the mechanism of energy homeostasis remodeling induced by blocked glucose uptake in fish.

## Materials and methods

### Ethics statement

Experimental zebrafish were obtained from the Institute of Hydrobiology, Chinese Academy of Sciences (Wuhan, Hubei, China). Animal experiments and treatments were performed according to the Guide for Animal Care and Use Committee of the Institute of Hydrobiology, Chinese Academy of Sciences (IHB, CAS, Protocol No. 2016–018).

### Zebrafish maintenance

All embryos were obtained by natural fertilization and were incubated in hatching water (4 L water + 6 mL sea salt + 200 μL methylene blue saturated solution) at 28.5°C with no more than 50 embryos per dish. Developmental stages were determined by days post-fertilization (dpf) or months post-fertilization (mpf) (27). Dead embryos were promptly removed at 0–3 dpf, and membranes were removed at 3 dpf. At 5 dpf, all larvae were transferred to standing water aquariums (50 larvae/aquarium/L) and fed with paramecia. From 9 to 15 dpf, they were fed with paramecia and a small amount of newly hatched brine shrimp (*Artemia cysts*) (Tianjin Fengnian Aquaculture Co., Ltd., Tianjin, China). From embryos to 15 dpf larvae, half of the rearing water was replaces daily. At 15 dpf, all larvae (60 fish/aquarium/10L) were transferred to a circulated water system and fed with newly hatched brine shrimp twice a day at 28.5°C under a 14-h light and 10-h dark photocycle.

### The analysis of *glut2* transcriptional expression

Six wild-type zebrafish (3 mpf) were exposed to ice bath anesthesia and their tissues were immediately separated into RNAlater™ solution (AM7020, Invitrogen, USA). The *glut2* mRNA tissue distribution analysis was then performed according to the previously described method (28).

### Establishment of *the glut2*-deletion zebrafish line

According to a previous study, *glut2*-knockout zebrafish were created using CRISPR/Cas9 technology (29). Briefly, male zebrafish with mosaic mutations (F0) were mated with wild-type zebrafish to create heterozygous F1 offspring; female fish were further purified with another control male to generate an F2 population. F3 fish, with *glut*2^+/+^(control), *glut*2^+/−^, and *glut*2^−/−^ genotypes, were obtained from crossing *glut2* F2 heterozygotes at 3 mpf. Examination of fish genotypes from the population was carried out as previously described (25). Two effective mutant lines were obtained, and the knockout efficiency was further verified using qPCR analysis, which was performed on 5 dpf F3 population zebrafish (control and *glut*2^−/−^) as previously described (30).

### Hatching rate and survival

Six pairs of male and female heterozygote F2 zebrafish (6 mpf) from the same parent were selected as parent fish to ensure that the genetic background of the experimental fish was similar. Then, 100 eggs of each pair were randomly selected and incubated to calculate the hatching rate: hatching rate = 100 × (100 – number of dead embryos – number of unbroken embryos until 3 dpf)/100. The survival rate of zebrafish was analyzed from 6 to 21 dpf. The dead zebrafish were timeously collected in PCR tubes and stored at −80°C. All live fish were killed in an ice bath and genotyped together with the dead fish. Here, 236 fish were successfully identified for survival analysis.

### Photographing and growth performance

Juvenile zebrafish at 30 dpf were anesthetized using MS-222 (Sigma, St Louis, MO, USA), and each fish was immediately photographed and genotyped. The images were imported into ImageJ software for body length measurement, which was standard with a known length bar. Each genotyped zebrafish was then weighed. Here, 105 fish were used for growth performance analysis.

### Hatching rate and survival of MZ*glut2* zebrafish

The homozygous fish (F3) could partially survive and self-cross to produce the maternal zygotic mutant *glut2* (MZ*glut2*) zebrafish (F4). To ensure that the genetic background of the next experimental fish was similar to the previous experimental fish, the F4 zebrafish (including the control group) used the same pair of male and female heterozygotes F2 zebrafish offspring as their parent fish (F3). The hatching rate and survival analysis were then performed as described above (200 larvae per genotype were used for survival analysis). Only male fish were analyzed in the following study to avoid ovulation cycle bias on lipid and glucose metabolism (31).

### Growth performance of MZ*glut2* zebrafish

Until their adult stage (3 mpf), fish (21 fish for each genotype) were randomly selected and anesthetized using MS-222, and the body length was measured, and the corresponding weight was weighted.

### Glucose measurement

After ice bath anesthesia, the caudal fin was severed with scissors and whole blood collected from the wound was immediately used for blood glucose measurement using a blood glucose meter (Nipro Diagnostics, Inc., Florida, USA). Liver and muscle tissues (two fish for one sample, eight replicates for each genotype) were then immediately harvested and their glucose content was measured according to the instructions of the assay kit (MS2601, Shanghai Cablebridge Biotechnology Co., Ltd., China).

### Glucose uptake assay

Hepatocyte preparation was performed according to the previous study (32). The control and MZ*glut2* zebrafish primary hepatocyte (12 fish per genotype at 3 mpf) were separated and incubated in DMEM/F12 medium at 28.5°C in 5% CO2 for 24 h. The medium was then discarded, and PBS was added to slowly wash the cells; the PBS was removed for the glucose uptake assay. The glucose uptake assay in hepatocytes was performed according to assay kit instructions (J13114, Promega, USA). Briefly, the cells were treated with 10 mM 2-deoxyglucose (2-DG) for 10 min at 28.5°C, and their luminescence intensity was measured according to the manufacturer's instructions.

### Dynamic detection of blood glucose

Glucose tolerance tests were performed on the control and MZ*glut2* fasting (16 h) zebrafish at 3 mpf. Their dynamic blood glucose levels were measured using a glucose meter after ice bath anesthesia using two methods (six fish for each time point). First, fasting zebrafish were immersed in a 3% glucose solution for 3 h, and following hyperglycemic induction, blood glucose concentrations at different time points were detected. Second, zebrafish were fed a high carbohydrate diet (40% dextrin) for 6 days before the experiment was started, and after the diet was consumed for 7 days, blood glucose concentrations were measured at different time points (calculated from the first bite of food). The caudal fin was severed with scissors after ice bath anesthesia, and whole blood collected from the wound was used for blood glucose measurement. The high carbohydrate diet used in this experiment is shown in Supplementary Table 1.

### qRT-PCR analysis

The qRT-PCR analysis was performed as per the detailed steps in our previous study (33). Briefly, total mRNA was extracted using TRIzol reagent according to the manufacturer's instructions (Invitrogen, Carlsbad, USA), and cDNA was synthesized using a cDNA synthesis kit (TransGen Biotech, AE311-03). A real-time quantitative PCR was performed using SYBR Green I Master Mix (Roche, Germany) on a Light-Cycler 480 system (Roche). All mRNA levels were calculated as fold expression relative to the housekeeping gene *rpl7*. The primers used for qPCR are listed in Table 1. In the present study, since *glut2* is thought to be a high Michaelis constant (Km) transporter, intestinal and liver tissue samples were taken at 2 h of high carbohydrate feeding to monitor glucose transporter expression. However, the difference is that we sampled lipid-related gene expression at 6 h after a normal meal, while protein-related gene expression was sampled at 24 h after a normal meal.

**Table 1:** Primers used in this study.

**Gene name**	**Primer direction^a^ and sequence (5′-3′)**	**Accession no**.	**Product size^b^**
**Genotype examination**			
*glut*2^c^	F: CAGATGGGATACAGCTTGG	NM_001042721.1	181
	R: AGATGGCGACGGATAAAGA		
**qPCR**			
*rpl*7^c^	F: CAGAGGTATCAATGGTGTCAGCCC	NM_213644.2	119
	R: TTCGGAGCATGTTGATGGAGGC		
*glut*2^c^	F: CCACCGAAAACATGGAGGAGTT	NM_001042721.1	167
	R: TGTCATAACACCTGGGCTCTGTG		
*glut*1^c^	F: TATTGGACGGTTTGTGGTG	XM_002662528.5	118
	R: AAGTTGATGAAGTGTGCCC		
*glut*3^c^	F: CACTGGAGAGCCGATGGATG	XM_002667123.5	135
	R: ATGGACTTCCGTCCTCCAAG		
*glut*5^c^	F: TGATGGGTGTGAGTGAAGTG	NM_001365652.1	297
	R: GGAAGAAAGGCAGAAGCAG		
*glut*8^c^	F: TGACCAGTGTGCTAACGGAC	NM_212798.1	300
	R: TAGAAGCACACAGTGCCGAG		
*glut*9^c^	F: CTTCGGCTTTTCAGCGATGG	XM_017359073.2	157
	R: CAACACAAACGGGACACCAC		
*glut*12^c^	F: GGGACAATCCTGGACCACTA	NM_200538.1	136
	R: ACATCCCAACCAGCATTCTC		
*sglt*1^c^	F: ATTGGAGCCTCTCTCTTCGC	NM_200681.1	177
	R: CATAGTCACAACCCCAGCCT		
*insra* ^c^	F: GCGTGGCAATAATCTGTTCT	NM_001142672.1	278
	R: CGTTGATAGTGGTGAGGGGG		
*insrb* ^c^	F: TTTCGCCTACATCTTGTGCC	NM_001123229.1	101
	R: AGTTCTCCAAAACCCGCA		
*chrebp* ^c^	F: ACCCCGACATGACCTTCAAC	NM_001328694.1	157
	R: TGTGGCATCTCTGTGTTGCT		
*srebf*1^c^	F: ATGGCGGAAGACAGCAA	NM_001105129.1	107
	R: AGCGGGTTAAAGGACAGAA		
*srebf*2^c^	F: CACACTCTTCTCTCTGCCCG	NM_001089466.1	165
	R: GATGTCGGTGAGTGAAGGGG		
*aclya* ^c^	F: GAGCTCCGAGTGAGCAACAA	NM_001002649.2	157
	R: AAAGCCCTGACGATACCCTTG		
*fasn* ^c^	F: GGAGCAGGCTGCCTCTGTGC	XM_009306807.3	128
	R: TTGCGGCCTGTCCCACTCCT		
*acc* ^c^	F: GCGTGGCCGAACAATGGCAG	XM_021476192.1	137
	R: GCAGGTCCAGCTTCCCTGCG		
*fads*2^c^	F: CAGCATCACGCTAAACCCAAC	NM_131645.2	164
	R: AGGGGAGGACCAATGAAGAAG		
*scd* ^c^	F: AGCCACTTTACCTCTGCG	NM_198815.2	219
	R: AGCTCTAGTTTGCGTCCT		
*dgat*1*a*^c^	F: CCAAAGCTCGAACCCTGTCT	NM_199730.1	104
	R: GTGTGTGAGGTTTCCCGGAT		
*dgat*2^c^	F: ACGCATAACCTGCTTCCC	NM_001030196.1	102
	R: TCCTGTGGCTTCTGTCCC		
*atgl* ^c^	F: CCTGCAAGGAGTGAGGTATG	XM_005174256.4	192
	R: CTGTAGAGGTTGGCGAGTGT		
*lpl* ^c^	F: GCTCTCACGAGCGCTCTATT	NM_131127.1	293
	R: CTTCATGGGCTGGTCAGTGT		
*pparab* ^c^	F: TCAGGATACCACTATGGCGTTCAT	NM_001102567.1	100
	R: AGCGGCGTTCACACTTATCGTA		
*cpt*1*aa*^c^	F: CATCCTTAGGCCTGCTCTTCAAA	NM_001044854.1	94
	R: ACCATGACACCCCCAACTAACAT		
*cpt1ab* ^c^	F: GACTTCCAATTACGTCAGCGA	XM_005170707.4	189
	R: TGTGCTCTGTCCAGTTTTCTCC		
*acox*3^c^	F: TGGAAGGACATGATGCGCTTT	NM_213147.1	102
	R: AGGCTGCCGGGCAAAAA		
*mtor* ^c^	F: TGGGAGCAGACAGGAATGAAGG	NM_001077211.2	97
	R: TGCACCTGCTGGAAAAAGAATG		
*bcat*1^c^	F: GGGCTCGTACTTCAGCACAGGA	NM_200064.1	104
	R: TCCCTCCCATCTTGCAGTCTCC		
*bcat2* ^c^	F: CCGACCATCGCTGTCCAGAATG	NM_001002676.2	100
	R: TCATGGTGCCGACCTCAGTGAT		
*bckdha* ^c^	F: TCCGACGAGAAGCCGCAGTT	NM_001024419.1	117
	R: GCCCTGTCTGTCCATCACTCTG		
*bckdk* ^c^	F: TTGATTTTGCTCGACGGCTCT	NM_213060.2	95
	R: TGGAATGAAGGGAAAGCGGG		
*glud*1*b*^c^	F: GATGTCCTGGATTGCTGACACCT	NM_199545.4	96
	R: CCACCCTGGCTAATGGGTTTT		
*asns* ^c^	F: TTCAGAATGCTGACTGACGATGG	NM_201163.3	105
	R: TGGAAAAGCAGTGATCTTTGCAG		
*atf*4*a*^c^	F: AGATGAGCACACTGAGGTTCCA	NM_213233.1	120
	R: TCGGAGCAATCGCTAATGTTCT		
*eif*4*ebp*3^c^	F: CCGCTTCCGGACAGTTACA	NM_001007354.2	78
	R: ATAGATAATCCGAGTTCCGCC		
*murf*1*a*^c^	F: AGCCTGTTGTCATTCTCCCG	NM_001002133.1	126
	R: CCTCGAAGCGACAAGTAGGG		

^a^F: Forward primer; R: Reverse primer. ^b^bp: base pair. ^c^*glut2*, solute carrier family 2 member 2; *rpl7*, ribosomal protein L7; *glut1*, solute carrier family 2, member 1; *glut1*, solute carrier family 2, member 1; *glut3*, solute carrier family 2, member 3; *glut5*, solute carrier family 2, member 5; *glut8*, solute carrier family 2, member 8; *glut9*, solute carrier family 2, member 12; *sglt1*, solute carrier family 5 member 1; *insra*, insulin receptor a; *insrb*, insulin receptor b; *chrebp*, carbohydrate-responsive element-binding protein-like; *srebf1*, sterol regulatory element binding transcription factor 1; *srebf2*, sterol regulatory element binding transcription factor 2; *aclya*, ATP citrate lyase a; *fasn*, fatty acid synthase; *acc*, acetyl-CoA carboxylase alpha; *fads2*, fatty acid desaturase 2; *scd*, stearoyl-CoA desaturase; *dgat1a*, diacylglycerol O-acyltransferase 1a; *dgat2*, diacylglycerol O-acyltransferase 2; *atgl*, adipose triglyceride lipase gene; *lpl*, lipoprotein lipase; *pparab*, peroxisome proliferator-activated receptor alpha b; *cpt1aa*, carnitine palmitoyltransferase 1Aa; *cpt1ab*, carnitine palmitoyltransferase 1Ab; *acox3*, acyl-CoA oxidase 3; *mtor*, mechanistic target of rapamycin kinase; *bcat1*, branched chain amino-acid transaminase 1; *bcat2*, branched chain amino-acid transaminase 2; *bckdha*, branched chain keto acid dehydrogenase E1 subunit alpha; *bckdk*, branched chain ketoacid dehydrogenase kinase; *glud1b*, glutamate dehydrogenase 1b; *asns*, asparagine synthetase; *atf4a*, activating transcription factor 4a; *eif4ebp3*, eukaryotic translation initiation factor 4E binding protein 3; *murf1a*, muscle-specific RING finger 1a.

### β-cell monitoring, proliferation, and whole-mount *in situ* hybridization (WISH) analyses

*Tg* (*insulin:EGFP*) (34) was used to mark β-cells for imaging and/or counting as previously described (35). The β-cells of control and MZ*glut2* zebrafish were monitored at 5 dpf. Fisetin was considered a GLUT2 inhibitor (36), and a concentration of 120 μm was used to incubate 3–5 dpf *Tg* (*insulin:EGFP*) embryos to monitor the β-cells at 5 dpf. Proliferation was analyzed as previously described (35). Briefly, the Click-iT EdU Alexa Fluor 594 Imaging kit (C10339, Invitrogen) was used to identify proliferating β cells. At 4 dpf, 1–2 nL of 0.1 mM 5-ethynyl-2-deoxyuridine (EdU) was injected into the fish heart. After 24 h, the fish were euthanized and fixed in 4% paraformaldehyde to detect the signals. WISH was performed as previously described (37). Antisense digoxigenin-labeled insulin cRNA was synthesized and used in this study.

### HM350 metabolome analysis

Control and MZ*glut2* zebrafish livers were sampled (three livers per sample, six samples per genotype) in a 1.5-mL tube, which immediately froze in liquid nitrogen. These samples were then sent to the Beijing Genomics Institution for HM350 metabolome analysis.

### Determination of lipids and crude proteins

Lipid and crude protein content was measured according to our previous descriptions (33). Briefly, zebrafish were freeze-dried and then ground using a mortar. Next, the chloroform/methanol (V/V, 2:1) extraction technique was used to measure the lipid content of zebrafish. Crude protein content (N × 6.25) was determined after acid digestion using an auto Kjeldahl system (Kjeltec-8400, FOSS Tecator, Haganas, Sweden).

### Nile red staining, triglyceride measurement, and Oil Red O staining analyses

Neutral lipid accumulation was visualized using fluorescent dye staining, Nile red, in live fish as previously described (25, 38). Nile red (N3013; Sigma) was dissolved to a concentration of 0.1 g/mL. The fish were immersed in Nile red at 28.5°C overnight in the dark. Images were taken using an Olympus SZX16 FL stereomicroscope (Olympus, Tokyo, Japan) at an excitation wavelength of 488 nm. Liver triglycerides were measured using commercially available kits (A110-1, Nanjing Jiancheng Bioengineering Institute, China). Fatty droplet accumulation in the liver was visualized using Oil Red O staining as previously described (25).

### Western blotting

A Western blot analysis was performed on the liver and muscle tissues of 12 zebrafish from each genotype (one sample mixed with tissues from four fish). The Western blot analysis protocols were performed using the methods described in our previous study (39). The primary antibodies of P-AMPK (1:1000; #2535S; Cell Signaling Technology, Danvers, MA, USA) and β-ACTIN (1:1000; #4970S; Cell Signaling Technology) were used in this study.

### Adenosine triphosphate content analysis

For liver and muscle ATP content analysis, 12 individual fish from each genotype were sampled (two fish tissues for one sample, six replicates for each genotype) and measured according to the instructions of the assay kit (A095-1-1, Nanjing Jiancheng Bioengineering Institute, China). The results were standardized using a protein concentration kit (A045-3, Nanjing Jiancheng Bioengineering Institute, China).

### Hematoxylin and eosin staining

For the muscle histological analysis, six individual fish from each genotype were fixed in 4% paraformaldehyde at 4°C for 24 h, followed by routine paraffin sectioning and H&E staining. The cross-section at the base of the cloaca was selected to quantify the total muscle area. Individual total muscle area was determined using the CaseViewer software.

### Locomotion tracking analysis

The locomotion tracking analyses were tracked and analyzed as previously described (40, 41). Briefly, after 6 h postprandial, each zebrafish was kept in a tank, and locomotion tracking was recorded for 5 min; the large speed distance and slow-mild speed distance were calculated in each tank for evaluation using the ZebraBox system (ViewPoint Life Sciences, Montreal, QC, Canada).

### Measurement of oxygen consumption

Oxygen consumption was measured and analyzed as previously described (40, 41). Briefly, four control or MZ*glut2* male fish were placed in an 1,150-mL conical flask and sealed for 6 h. Dissolved oxygen before and after sealing was measured using a dissolved oxygen respirator (41). Oxygen consumption was calculated as oxygen consumption per unit body weight. The oxygen consumption of 6 h postprandial (hpp) was calculated from 6 h postprandial fish fed with regular food. Basic oxygen consumption was calculated from the fish after starvation and lasted for 2 days. Four groups of each genotype were set as replicates.

### Statistical analysis

Statistical significance was determined using a two-tailed unpaired Student's *t*-test. Statistical analyses were performed using GraphPad Prism 8.0.1 (GraphPad Software, San Diego, CA, USA). All results are presented as mean ± SEM (standard error of the mean). A *P*-value of < 0.05 was considered statistically significant.

## Results

### *glut*2^−/−^ zebrafish from the F3 population showed a 30% survival rate and stunted growth

**Figure 1:**
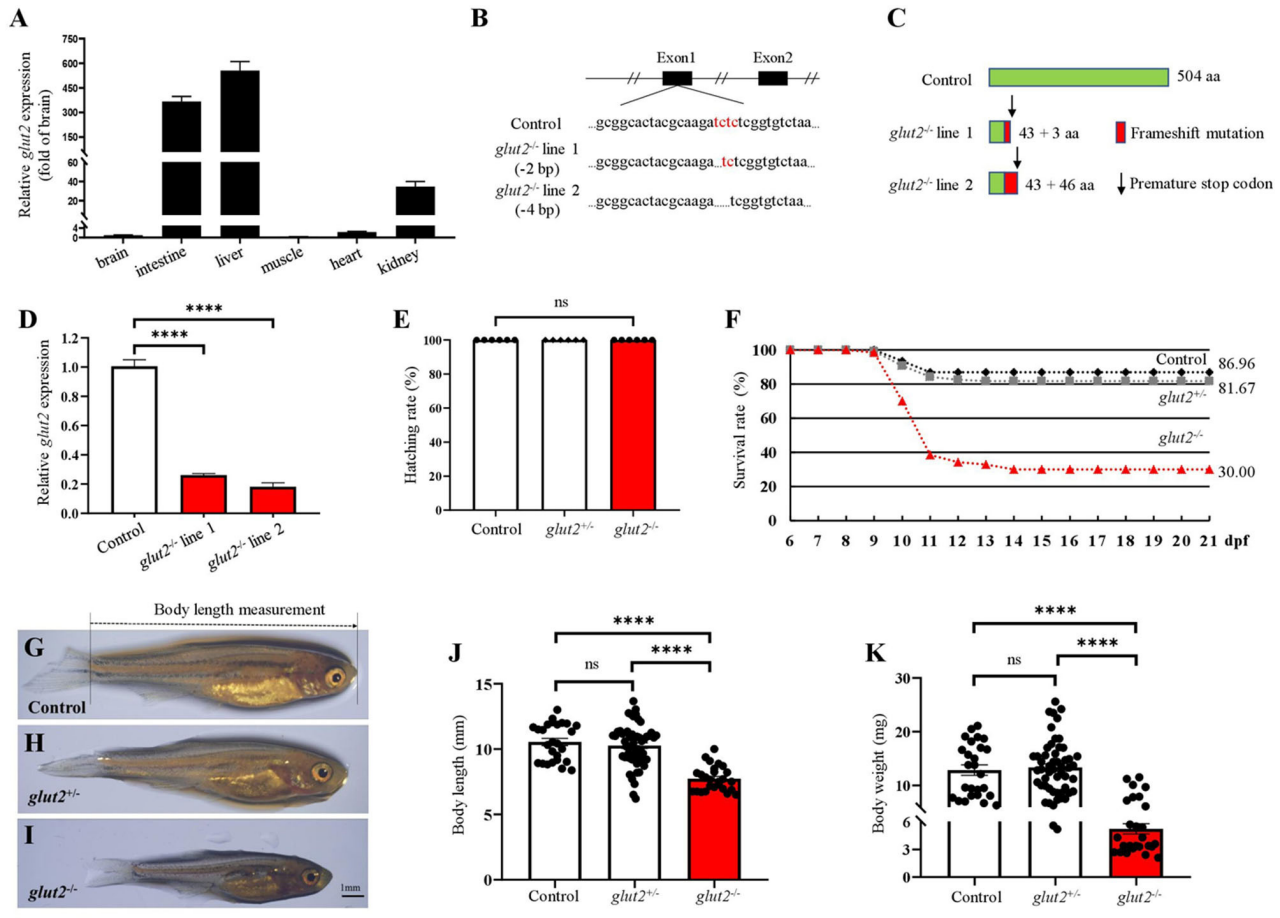
The knockout of *glut2* in zebrafish. **(A)** Transcriptional expression analysis of *glut2* in different tissues (*n* = 6). **(B)** Sequence comparison of *glut2* alleles in the control and two *glut2* knockout zebrafish lines. **(C)** Schematic representation of *glut2* full-length putative peptide from the control and two *glut2* knockout fish lines. aa, amino acids. **(D)** The transcriptional expression of *glut2* in 5 dpf control and *glut*2^−/−^ fish in the F3 population of both lines 1 and 2 (*n* = 3). **(E)** The hatching rate analysis of the control (*glut*2^+/+^), *glut*2^+/−^, and *glut*2^−/−^ fish from 0–3 dpf in the F3 population of line 2 (*n* = 6). **(F)** Analysis of the control, *glut*2^+/−^, and *glut*2^−/−^ survival rates from 6 to 21 dpf in line 2 F3 population (236 fish were genotyped for analysis). **(G–I)** Representative images of the overall morphology of the control, *glut*2^+/−^, and *glut*2^−/−^ fish at 30 dpf. (**J, K**) Body weight and body length of the control (*n* = 26), *glut*2^+/−^ (*n* = 53), and *glut*2^−/−^ (*n* = 26) fish at 30 dpf. A two-tailed unpaired *t*-test was used to detect significance. ****P < 0.0001. ns, no significance.

We first examined *glut2* transcriptional levels in different zebrafish tissues. The abundant transcriptional expression of *glut2* was observed in the liver, intestine, and kidney, suggesting its physiological role associated with glucose uptake in target organs where *glut2* is highly expressed (Figure 1A). The putative mRNA of *glut2* is 1515 base pairs in length and encodes 504 amino acids. Using CRISPR/Cas9 technology, *glut2* knockout in zebrafish with target site mutation was established by microinjection of recombinant Cas9 protein and gRNA from *glut2* target sites. The two knockout lines with 2 and 4 base pair deletions were obtained (Figure 1B). PCR products with genomic DNA and cDNA were used as templates for mutation validation. Premature stopping occurs in *glut*2^−/−^ fish from both knockout lines, which both retain 43 correct amino acids (Figure 1C). Transcriptional expression of *glut2* in the 5 dpf control and *glut*2^−/−^ fish in both line one and line two F3 populations was examined. The downregulated transcriptional expression of *glut2* in *glut*2^−/−^ fish compared to control fish indicates knockout efficacy (*P* < 0.05) (Figure 1D); this is usually considered due to nonsense-mediated mRNA decay. In the F3 population of line two, comparable hatching rates of *glut*2^+/+^, *glut*2^+/−^, and *glut*2^−/−^ were observed from 0 to 3 dpf (*P* > 0.05) (Figure 1E). Subsequently, the F3 population survival rate was statistically analyzed, and a significant decrease was observed in *glut*2^−/−^ fish, as they died sharply from 9 to 14 dpf (Figure 1F). Intriguingly, approximately 30% of *glut*2^−/−^ fish survived to adulthood and could reproduce. Compared to *glut*2^+/+^ and *glut*2^+/−^ zebrafish at 30 dpf, the surviving *glut*2^−/−^ fish exhibited apparent growth retardation, shortened body length, and decreased body weight observed (*P* < 0.05) (Figures 1G–K).

**Figure 2:**
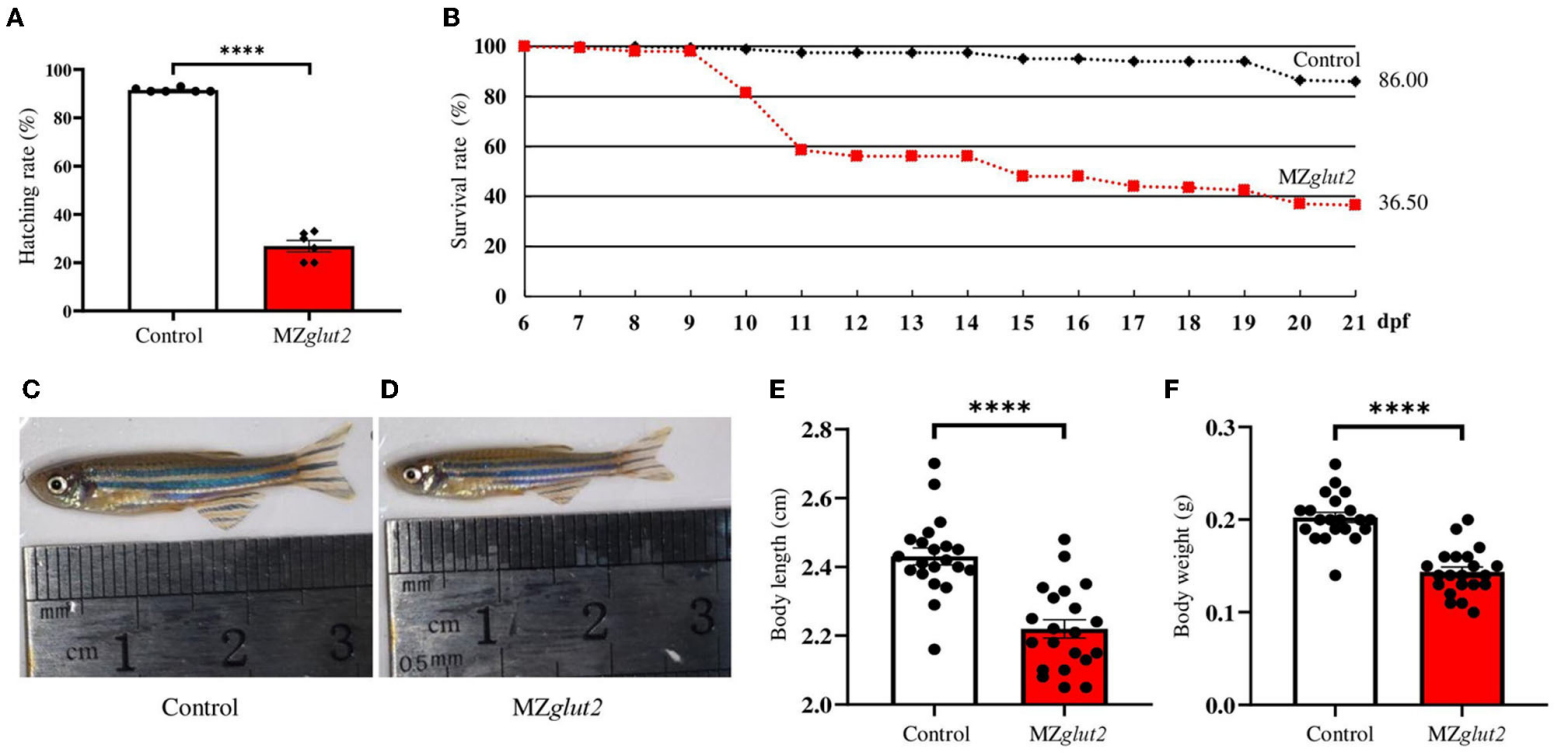
*glut2*-deletion results in growth retardation in MZ*glut2* fish. **(A)** The hatching rate analysis of the control and MZ*glut2* fish from 0 to 3 dpf (*n* = 6). **(B)** Survival rate analysis of the control and MZ*glut2* fish from 6–21 dpf (200 fish of each genotype were used for analysis). **(C, D)** Representative images of the overall morphology of the control and MZ*glut2* fish at 3 mpf. **(E, F)** Body weight and body length of the control and MZ*glut2* fish at 3 mpf (*n* = 21). A two-tailed unpaired *t*-test was used to detect significance. ****P < 0.0001.

Subsequently, the hatching rate and growth performance of control (offspring from natural mating of *glut*2^+/+^ males and females) and MZ*glut2* fish were compared and analyzed. Hatching rates of MZ*glut2* fish decreased significantly compared to control fish (*P* < 0.05) (Figure 2A). This result suggested that maternal *glut2* is essential and critical for hatching. Similarly, more than 60% lethality occurred in MZ*glut2* fish up to 21 dpf, as well as arrested growth performance at 3 mpf (e.g., decreased body length and weight (*P* < 0.05) (Figures 2B–F).

### Glucose uptake was effectively inactivated in MZ*glut2* fish

**Figure 3:**
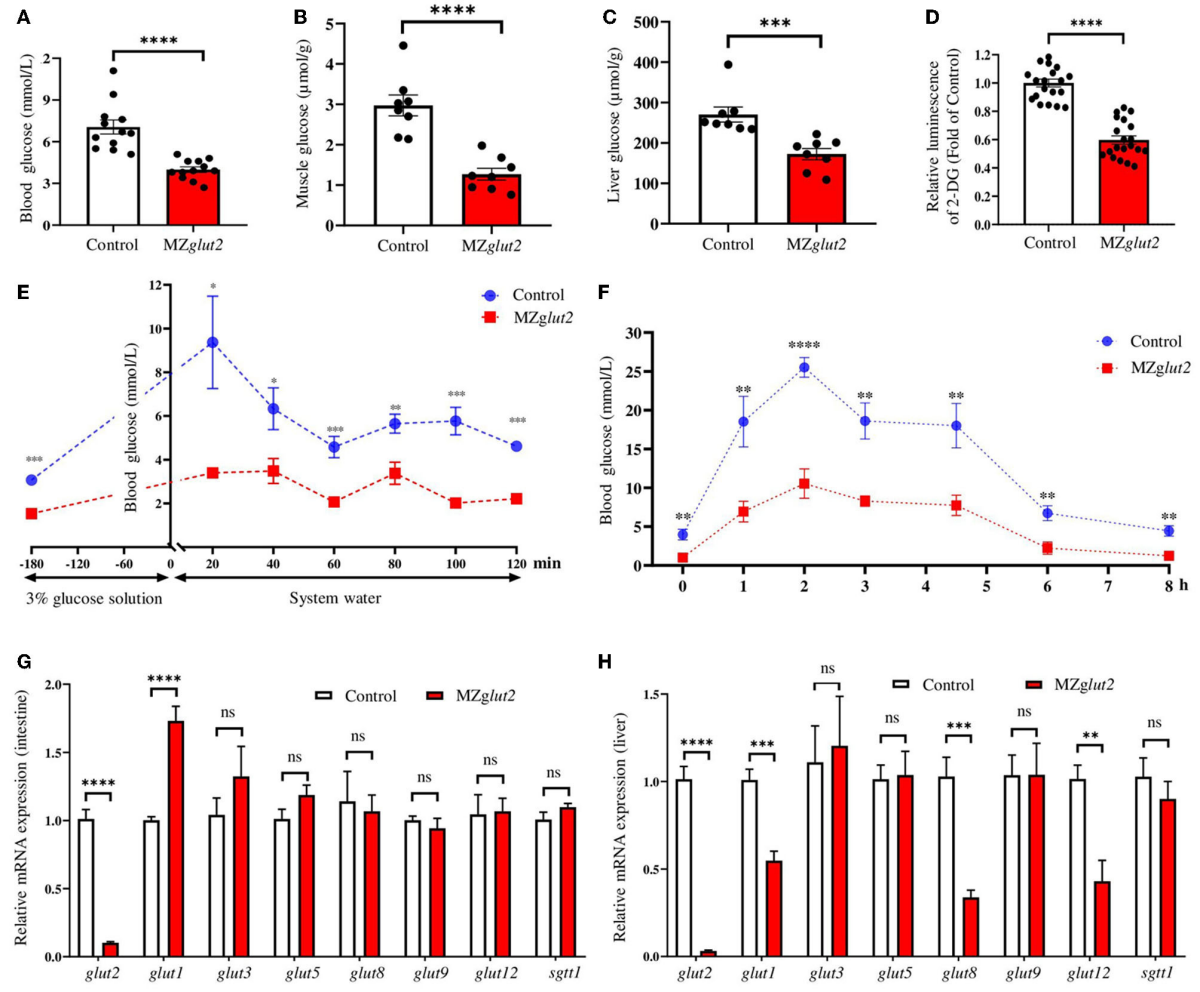
*glut2*-deletion results in impaired glucose uptake in MZ*glut2* fish. **(A)** Glucose content in the blood of the control and MZ*glut2* fish (*n* = 12). **(B)** Glucose content in the muscle of the control and MZ*glut2* fish (*n* = 8). **(C)** Glucose content in the liver of the control and MZ*glut2* fish (*n* = 8). **(D)** The hepatic glucose uptake levels (2-DG) of the control and MZ*glut2* fish (*n* = 20). **(E)** Blood glucose levels of the control and MZ*glut2* fish after the glucose tolerance test for 20, 40, 60, 80, 100, and 120 min (*n* = 6). **(F)** Blood glucose levels of the control and MZ*glut2* fish after being fed a high carbohydrate diet for 0, 1, 2, 3, 4.5, 6, and 8 h (*n* = 6). **(G)** Transcriptional expression of *glut2, glut1, glut3, glut5, glut8, glut9, glut12*, and *sglt1* in the intestine of the control and MZ*glut2* fish after fed high carbohydrate diet at 2 h (*n* = 6). **(H)** Transcriptional expression of *glut2, glut1, glut3, glut5, glut8, glut9, glut12*, and *sglt1* in the liver of the control and MZ*glut2* fish after fed high carbohydrate diet at 2 h (*n* = 6). A two-tailed unpaired *t*-test was used to detect significance. ****P < 0.0001. ***P < 0.001. **P < 0.01. *P < 0.05. ns, no significance.

Adult control and MZ*glut2* (3 mpf) zebrafish were used for the following analyses. Compared to control fish, MZ*glut2* fish at 2 hpp showed decreased glucose levels in the blood, muscle, and liver (*P* < 0.05) (Figures 3A–C). The *in vitro* 2-DG uptake assay demonstrated that the 2-DG uptake decreased in the liver of MZ*glut2* fish compared to the control fish (*P* < 0.05) (Figure 3D). After administration of a 3% glucose solution for 3 h, significant increases in blood glucose were observed in the control and MZ*glut2* fish at 20 min after 3% glucose deprivation; however, MZ*glut2* fish blood glucose decreased before and after 3% glucose administration at all time points examined (*P* < 0.05) (Figure 3E). Dynamic blood glucose was also assessed after a high carbohydrate diet. Blood glucose peaked at 2 hpp in both control and MZ*glut2* fish, but the MZ*glut2* fish blood glucose decreased at all time points (*P* < 0.05) (Figure 3F). The compensatory effect of glucose transporters in the intestine of the MZ*glut2* fish at 2 hpp of the high carbohydrate diet was detected, as intestinal *glut1* transcriptional expressions were upregulated compared to control fish at 2 hpp (*P* < 0.05) (Figure 3G). Compensatory expression of other glucose transporters in the liver was not observed, as expressions of *glut2, glut1, glut8*, and *glut12* were downregulated in MZ*glut2* fish at 2 hpp of the high carbohydrate diet (*P* < 0.05) (Figure 3H). These results suggest that glucose uptake was effectively inactivated in MZ*glut2* fish.

### *glut2*-deletion results in impaired insulin-mediated anabolic metabolism in MZ*glut2* fish

**Figure 4:**
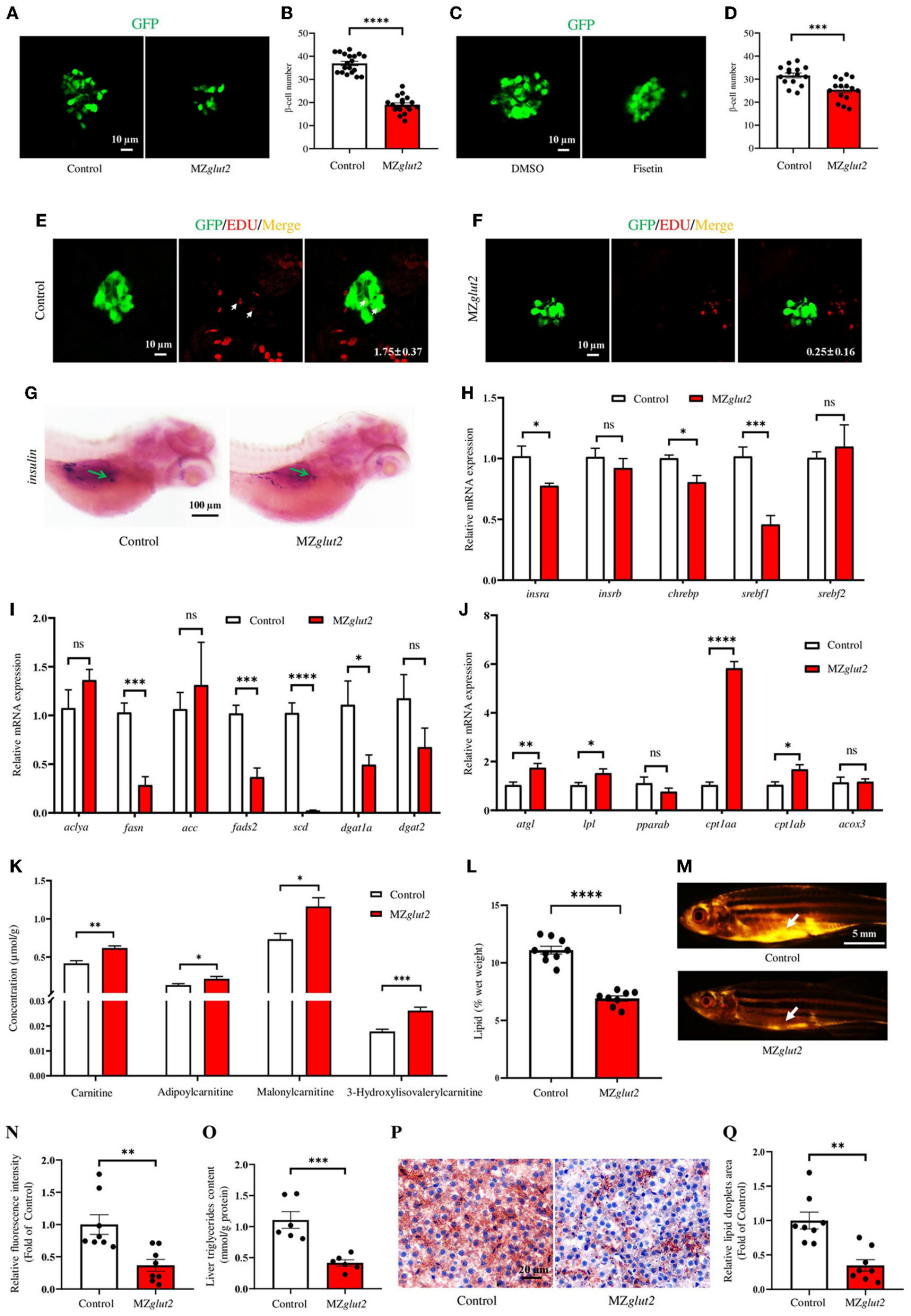
*glut2*-deletion results in impaired insulin-mediated anabolic metabolism in MZ*glut2* fish. **(A)** Representative images of β-cells in the control and MZ*glut2* larvae at 5 dpf. **(B)** The statistical analysis of β-cells in the control (*n* = 19) and MZ*glut2* (*n* = 18) larvae at 5 dpf. **(C)** Representative images of β-cells in DMSO- and Fisetin-treated larvae at 5 dpf. **(D)** The statistical analysis of β-cells in DMSO- and Fisetin-treated larvae at 5 dpf (*n* = 16). **(E)** Representative images of EdU stained β-cells in the control fish at 5 dpf. Red signal-positive β-cells are indicated by arrows (*n* = 8). **(F)** Representative images of EdU staining β-cells in MZ*glut2* larvae at 5 dpf (*n* = 8). **(G)** WISH using the insulin probe to the control and MZ*glut2* larvae at 5 dpf, with the green arrow representing the signal area. **(H)** The transcriptional expression levels of *insra, insrb, chrebp, srebf1*, and *srebf2* in the liver of the control and MZ*glut2* fish at 3 mpf (*n* = 6). **(I)** The transcriptional expression levels of *aclya, fasn, acc, fads2, scd, dgat1a*, and *dgat2* in the liver of the control and MZ*glut2* fish at 3 mpf (*n* = 6). **(J)** The transcriptional expression levels of *atgl, lpl, pparab, cpt1aa, cpt1ab*, and *acox3* in the liver of the control and MZ*glut2* fish at 3 mpf (*n* = 6). **(K)** The quantification of carnitine metabolites based on the observations of metabolomics in the liver of control and MZ*glut2* fish at 3 mpf (*n* = 6). **(L)** The measurement of the lipid content of the control (*n* = 9) and MZ*glut2* (*n* = 8) fish at 3 mpf. **(M)** Nile Red staining of the control and MZ*glut2* fish at 3 mpf. White arrows: visceral adipose tissue. **(N)** The bar chart represents the relative fluorescence intensity of the control and MZ*glut2* fish after Nile Red staining (*n* = 8). **(O)** Liver triglyceride content at 3 mpf (*n* = 6). **(P)** Oil Red O staining of liver sections for distribution of lipid droplets in the control and MZ*glut2* fish at 3 mpf. **(Q)** The bar chart represents the relative lipid droplet areas of control and MZ*glut2* fish after Oil Red O staining (*n* = 8). A two-tailed unpaired *t*-test was used to detect significance. ****P < 0.0001.***P < 0.001. **P < 0.01. *P < 0.05. ns, no significance.

To visualize and analyze the number of β-cells and insulin content in *glut2*-deletion fish, the transgenic line *Tg* (*insulin:EGFP*) was bred with the *glut*2^−/−^ fish. This enables us to obtain the *Tg* (*insulin:EGFP*);*glut*2^+/−^ fish, which were inbred to generate *Tg* (*insulin:EGFP*);*glut*2^−/−^ fish. The *Tg* (*insulin:EGFP*);MZ*glut2* fish from the natural mating of *Tg* (*insulin:EGFP*);*glut*2^−/−^ males and females were used for the β-cells visualization and analysis. Compared with the control fish at 5 dpf, the number of β-cells significantly decreased in MZ*glut2* fish (*P* < 0.05) (Figures 4A, B). Fisetin, which has a hypoglycemic effect (42), was administered to wild-type embryos. In wild-type fish at 5 dpf, the Fisetin-treated fish also displayed decreased number of β-cells (*P* < 0.05) (Figures 4C, D), mimicking a reduced β-cells number of MZ*glut2* fish. The EdU staining in control fish and MZ*glut2* fish at 5 dpf was carried out for 24 h, and the statistical analysis of EdU stained β-cells in MZ*glut2* larvae was decreased (*P* < 0.05) (Figures 4E, F). These observations were also supported by the WISH results, from which, the decreased *insulin* signal in MZ*glut2* fish at 5 dpf was detected (Figure 4G).

A downregulated insulin receptor gene (*insra*) was found in the liver of MZ*glut2* fish at 3 mpf (*P* < 0.05) (Figure 4H). Meanwhile, fatty acid synthesis (*chrebp, srebf1, fasn, fads2*, and *scd*) and triglyceride synthesis (*dgat1a*) were also downregulated (*P* < 0.05), but upregulated lipolysis genes (*atgl* and *lpl*) and fatty acid β-oxidation (FAO) (*cpt1aa* and *cpt1ab*) were observed in MZ*glut2* fish liver at 3 mpf (*P* < 0.05) (Figures 4H–J). We performed a metabolomic analysis of the liver to obtain an overview of their energy metabolism due to impaired glucose uptake. Carnitines are effective factors to lower lipid content, as they function as key transporters of fatty acids to mitochondria for FAO (43). For metabolite measurements, increased carnitine, adipoylcarnitine, malonylcarnitine, and 3-hydroxylisovalerylcarnitine metabolites were observed in the liver of MZ*glut2* fish (*P* < 0.05) (Figure 4K). We observed a significant decrease in lipid content in MZ*glut2* fish, as evidenced by lipid measurements (*P* < 0.05) (Figure 4L) and observations of neutral lipids with Nile red staining (*P* < 0.05) (Figures 4M, N). The biochemical measurement to examine the quantity of liver triglyceride was performed. The MZ*glut2* fish liver triglyceride content significantly decreased compared with the control fish (Figure 4O). Pathological features of the control and MZ*glut2* fish liver were examined with histological analysis and Oil Red O staining, from which, the significantly decreased fatty droplet accumulation was observed in MZ*glut2* fish liver at 3 mpf (*P* < 0.05) (Figures 4P, Q). These results suggest that *glut2*-deletion impaired insulin-mediated anabolic metabolism in MZ*glut2* fish.

### Glucose uptake attenuation enhanced AMPK-mediated catabolic metabolism in MZ*glut2* fish

**Figure 5:**
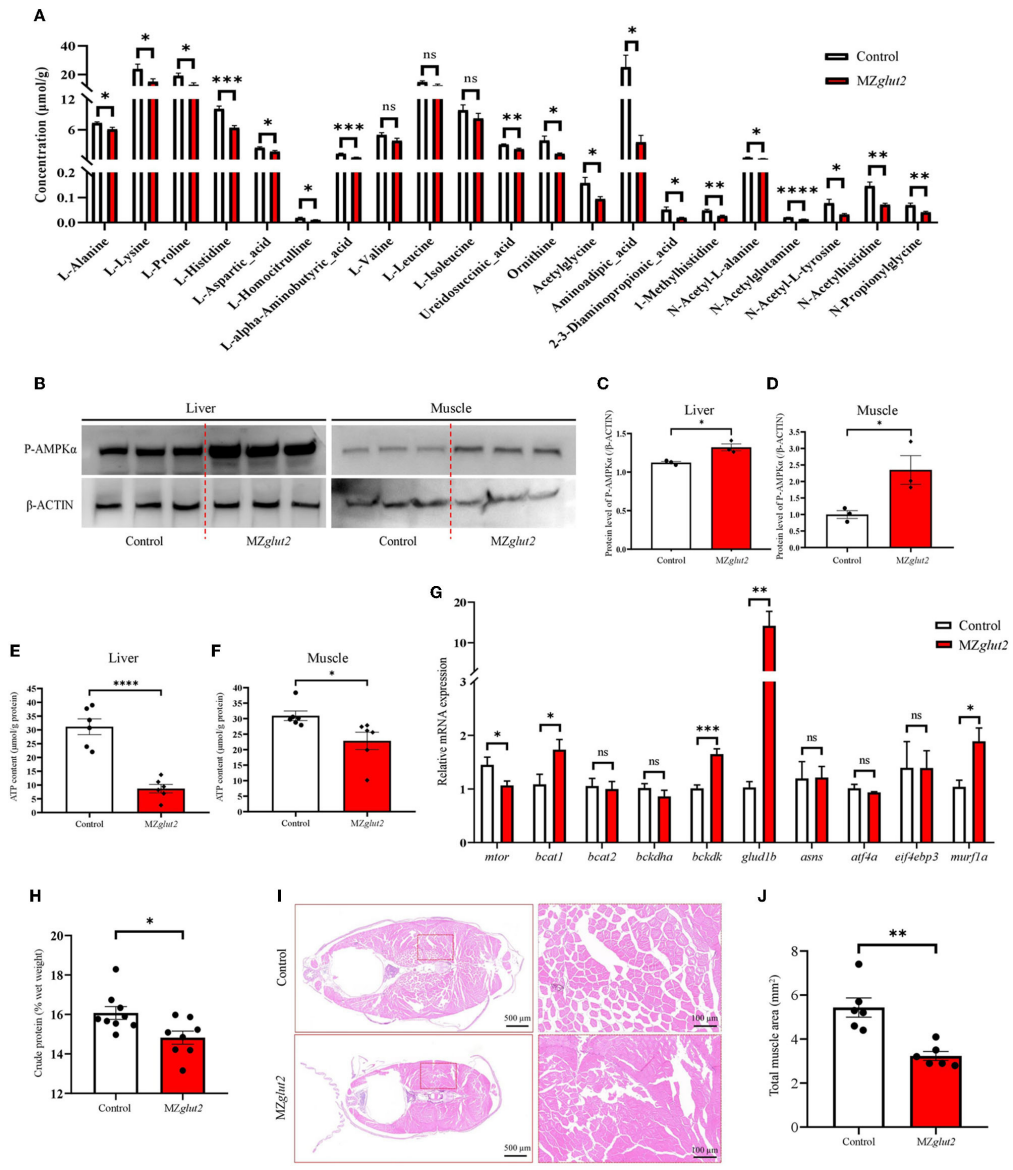
*glut2*-deletion results in enhanced AMPK-mediated catabolic metabolism in MZ*glut2* fish. **(A)** The quantification of amino acid metabolites is based on the observations of metabolomics in the liver of the control and MZ*glut2* fish at 3 mpf (*n* = 6). **(B–D)** The level of P-AMPK and β-ACTIN proteins in the liver and muscle of the control and MZ*glut2* fish (*n* = 3). **(E)** The ATP content in the liver of control and MZ*glut2* fish (*n* = 6). **(F)** The ATP content in the muscle of the control and MZ*glut2* fish (*n* = 6). **(G)** The transcriptional expression levels of *mtor, bcat1, bcat2, bckdha, bckdk, glud1b, asns, atf4a, eif4ebp3*, and *murf1a* in muscle of control and MZ*glut2* fish at 3 mpf (*n* = 6). **(H)** The measurement of the crude protein content of the control (*n* = 9) and MZ*glut2* (*n* = 8) fish at 3 mpf. **(I)** Representative images of H&E staining of the control and MZ*glut2* fish at 3 mpf. **(J)** The bar chart represents the total area of muscle mass in the body cross-section (*n* = 6). A two-tailed unpaired *t*-test was used to detect significance. ****P < 0.0001.***P < 0.001. **P < 0.01. *P < 0.05. ns, no significance.

Through metabolomics analysis, we found reduced amino acids, such as L-alanine, L-lysine, and L-proline, in the liver of MZ*glut2* fish (*P* < 0.05) (Figure 5A). AMPK is known as an energy sensor for activating lipid and protein catabolic metabolism when glucose is deficient. P-AMPK protein levels in the liver and muscle were significantly increased in MZ*glut2* fish at 24 hpp compared to control fish (*P* < 0.05) (Figures 5B–D), suggesting a low energy status of MZ*glut2* fish. Significantly reduced ATP levels were found in the liver and muscle of MZ*glut2* fish (*P* < 0.05) (Figures 5E, F). These results provided direct evidence that when glucose uptake was inactivated, insufficient energy supply from glucose activated catabolic metabolism. The *bckdk, glud1b*, and *murf1a* genes are known to activate amino acid catabolism and promote ubiquitin-mediated protein degradation (44, 45). All these genes were upregulated in MZ*glut2* fish muscle (*P* < 0.05), suggesting an increase in protein and amino acid degradation (Figure 5G). The downregulated expression of *mtor* in MZ*glut2* fish muscle not only reflects its attenuated protein synthesis but also agrees with the rise in proteolytic genes. These observations correlated with the decreased crude protein content (*P* < 0.05) (Figure 5H) and muscle mass (*P* < 0.05) (Figures 5I, J), which ultimately accounted for growth retardation.

### *glut2*-deletion resulted in slower movement activity but increased oxygen consumption in MZ*glut2* fish

**Figure 6:**
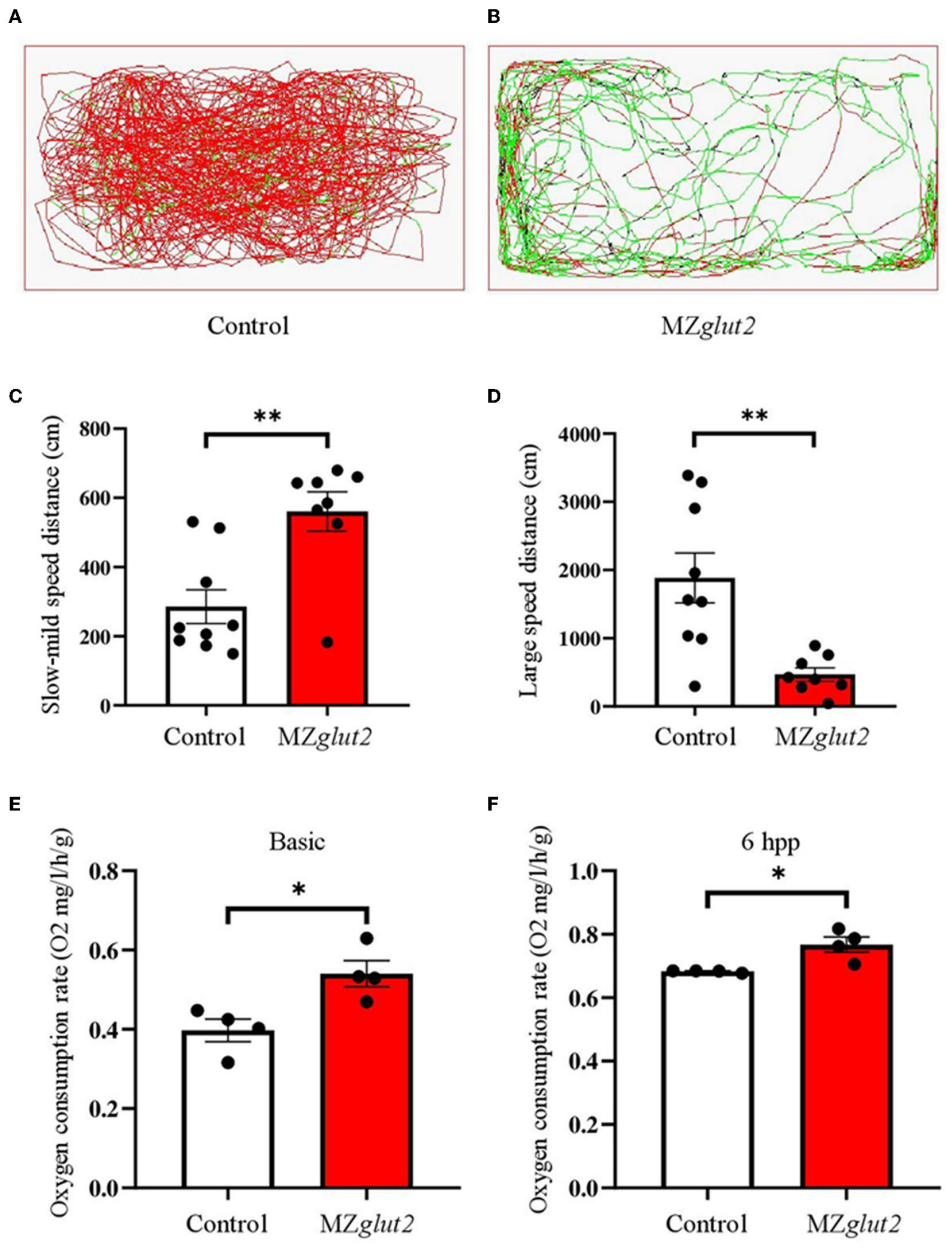
*glut2*-deletion resulted in slower movement activity but increased oxygen consumption in MZ*glut2* fish. **(A, B)** Pathway monitoring of the control and MZ*glut2* fish continued for 5 min at 3 mpf. **(C, D)** The distance covered with slow-mild speed or large speed of the control fish (*n* = 9) and MZ*glut2* (*n* = 8). Slow-mild speed (0–2 cm/s) and large speed (> 2 cm/s) of the control and MZ*glut2* fish. **(E, F)** The oxygen consumption rate of basic level and 6 hpp in the control and MZ*glut2* fish at 3 mpf (*n* = 4). A two-tailed unpaired *t*-test was used to detect significance. **P < 0.01. *P < 0.05.

The swimming activity of the control and MZ*glut2* fish was recorded for 5 min at 6 hpp. The distance traveled by MZ*glut2* fish was significantly reduced compared to the control fish (Figures 6A, B). Statistical analysis revealed that the slow-mild movement of MZ*glut2* fish was upregulated, while the large movement of MZ*glut2* fish was significantly downregulated (*P* < 0.05) (Figures 6C, D). To assess energy expenditure, we tested oxygen consumption in control and MZ*glut2* fish. Both basic and 6 hpp oxygen consumption was significantly increased in MZ*glut2* fish (*P* < 0.05) (Figures 6E, F).

**Figure 7:**
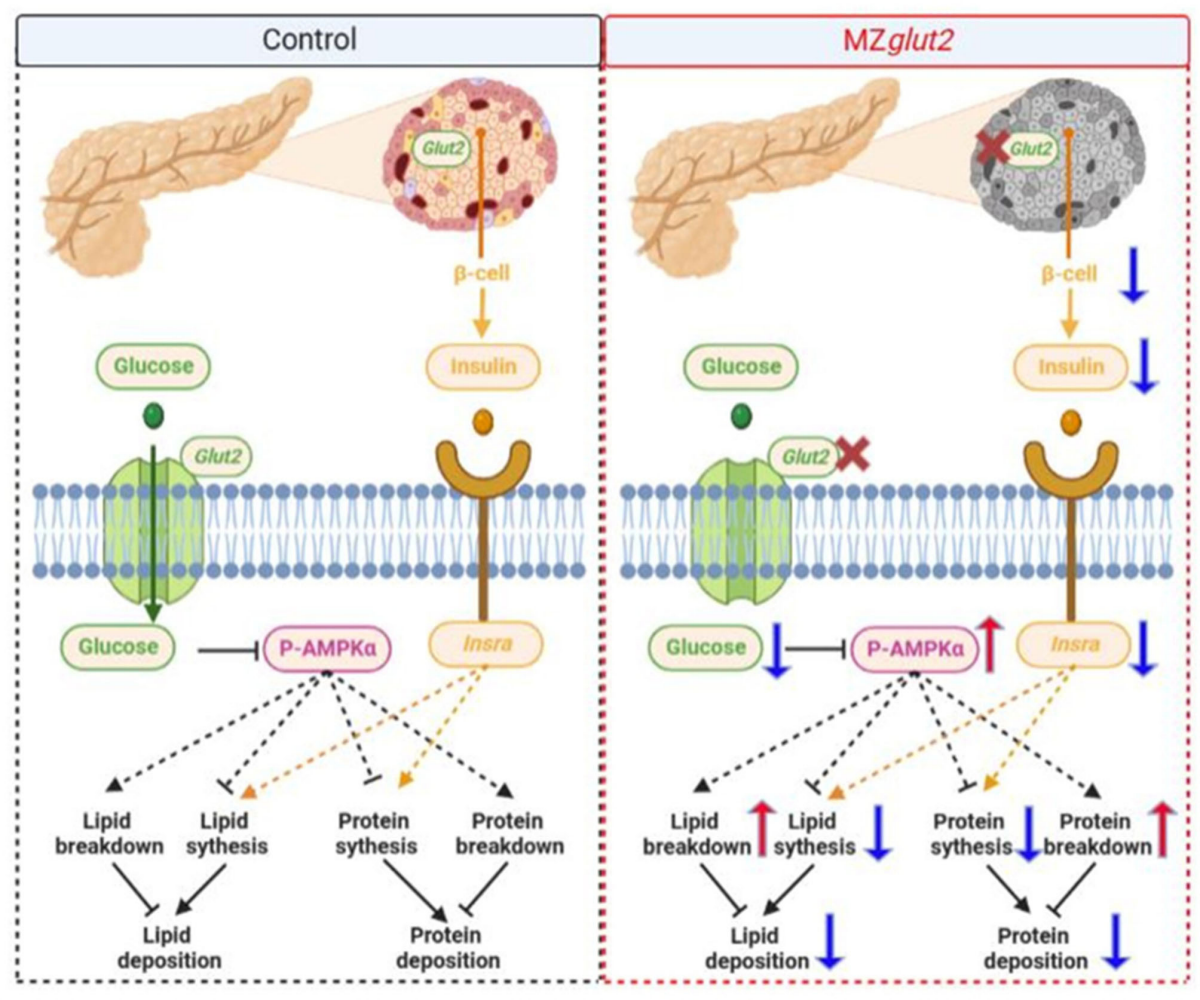
A hypothetical diagram showing how *glut2*-deletion induces remodeling of nutrient metabolism in zebrafish (the blue arrow means downregulation and the red arrow means upregulation).

Together, we found that *glut2*-deletion caused a decrease in the number of β-cells, which was related to the decrease of insulin-associated anabolic metabolism in surviving zebrafish. However, surviving zebrafish may adapt to blocked glucose uptake by remodeling the metabolic pattern *in vivo* by reducing insulin-mediated anabolism and enhancing AMPK-mediated catabolism (Figure 7).

## Discussion

Fish are known to have a poor ability to use dietary carbohydrates. Several metabolic disorders in fish were characterized to be caused by overloaded carbohydrates, as revealed by the high carbohydrate diet challenge undertaken in several studies (1–6). Regardless, the metabolic consequences of blocking glucose uptake under normal conditions are poorly investigated; therefore, the energy homeostasis remodeling of fish after blocking glucose uptake and the regulation of insulin and/or AMPK signaling are still unknown. The dynamic balance among glucose, fatty acids, and amino acids is an important prerequisite for maintaining cellular energy homeostasis (46). Glucose, which is absorbed through various GLUTs in different tissues, is the main source of metabolic energy for most cells (47). GLUT2 controls glucose uptake and glucose sensing, which is mainly expressed in the intestine, liver, and pancreatic β-cells (17). However, energy homeostasis remodeling after glucose uptake and *glut2* function in fish is still unknown. In the present study, the effects of energy homeostasis remodeling induced by blocking glucose uptake through *glut2*-deletion in zebrafish were characterized. We found abundant *glut2* expression in the liver and intestine of wild-type zebrafish, and *glut2*-deletion successfully blocked glucose uptake in zebrafish, which was validated as a useful tool for investigations to help discover the functions of glucose in fish.

In the present study, growth retardation was observed after glucose blocking by knocking out *glut2*, which was consistent with mutations in *Glut2* in mammals (17, 48). The growth of vertebrates is regulated by insulin signaling because insulin is known as the strongest anabolic hormone, which promotes the body's synthesis of proteins and lipids (25, 49). Insulin is secreted by pancreatic β- cells (50), and GLUT2 is thought to be one of the key factors in glucose-stimulated insulin secretion (17). Here, we found that *glut2*-deletion in zebrafish resulted in a decrease in β-cell number, which was caused by reduced proliferation. Similarly, the β-cell reduction was observed in *Glut2* knockout mice (20), which indicated a conserved *glut2* function for maintaining normal β-cell numbers between mice and zebrafish. Secreted insulin binds to insulin receptors to control downstream signal activation (30). In zebrafish, two insulin receptors play distinct roles in mediating glucose metabolism through the insulin signaling pathway (25). Based on our previous observations, *insra* is more likely to promote lipid synthesis, while *insrb* is more likely to promote lipid utilization and protein synthesis (25). In the present study, decreased *insulin* and *insra* expression were found in *glut2*-deletion zebrafish, indicating that the declined lipid synthesis may be mediated by impaired *Insulin/Insra* signaling, which is also supported by the downregulated lipid synthesis gene expression (*chrebp, srebf1, fasn*, etc.). Compared with the decrease in lipid content (*P* < 0.0001) in MZ*glut2* fish, decreased protein content (*P* < 0.05) and downregulated *mtor* (protein synthesis-related gene) were observed. Though *insrb* was not altered in MZ*glut2* fish, attenuated *Insulin*/*Insrb* signaling cannot be disregarded, which may be caused by the diminished insulin expression in β-cells. In summary, we concluded that *glut2*-deletion may cause impaired insulin signaling associated with attenuated anabolic metabolism in zebrafish.

As a prominent intracellular energy sensor, AMPK is directly activated by glucose deprivation (51). In this study, increased P-AMPK protein levels in the liver and muscle of MZ*glut2* fish indicated a poor energy status due to blocked glucose uptake. This was also directly reflected by glucose levels in the liver and muscle. In mice, liver-specific *Glut2* knockout inhibited liver glucose uptake (52), which is consistent with our results in the MZ*glut2* fish liver. However, energy homeostasis remodeling after glucose deprivation was not explored. The functions of AMPK in regulating metabolism are divided into two categories: inhibition of anabolism to reduce ATP consumption and stimulation of catabolism to increase ATP production (51). When cells have poor nutrition, catabolic pathways are activated, but energy-consuming biosynthesis of fatty acids and cholesterol is switched off (53, 54). In our study, switching on the catabolic pathways of lipolysis (*atgl* and *lpl*) and FAO (*cpt1aa* and *cpt1ab*), and switching off biosynthesis of fatty acids (*chrebp, srebf1, fasn, fads2*, and *scd*) and triglycerides (*dgat1a*) were observed in MZ*glut2* fish. These results indicated that AMPK-mediated inhibition of anabolism and stimulation of catabolism occurred in MZ*glut2* fish. AMPK activation directly promotes lipolysis and FAO, while directly inhibiting transcription factors such as carbohydrate-responsive element binding protein (ChREBP) and sterol regulatory element binding protein (SREBP), and other factors mediated lipid synthesis (51). Previously, Li et al. demonstrated that mitochondrial FAO inhibition resulted in energy homeostasis remodeling, as evidenced by the promotion of glucose utilization in fish (10, 12). However, repressed insulin signaling increased lipolysis and FAO also indicated energy homeostasis remodeling from glucose to lipid in MZ*glut2* fish. Lipids conserve protein for development more effectively than any other nutrient in fish (55). Thus, appropriate amounts of lipids can reduce protein breakdown in fish, replenishing the body's energy. However, we observed significant reductions in both lipid and protein contents after blocking glucose intake, indicating that lipids are no longer sufficient to maintain a normal energy supply. These findings could be supported by the enhanced amino acid breakdown (*bckdk, glud1b*, and *murf1a*) in muscle. Furthermore, protein degradation caused by blocking glucose uptake indicated that appropriate glucose uptake is essential for fish. Overall, elevated carnitines correlated with decreased protein and lipid deposition phenotype, which could be explained by the upregulated transcriptional expression of genes involved in protein and amino acid degradation, lipolysis, and FAO. Therefore, we proposed that *glut2*-deletion may have activated AMPK signaling associated with catabolic metabolism in zebrafish via insufficient ATP production. This hypothesis is supported by the reduced ATP content in the liver and muscle of MZ*glut2* fish, which was reflected by the decreased motility, increased protein levels of P-AMPK, and transcriptional expression of the above-mentioned genes in MZ*glut2* fish.

The complete lethality observed in *Glut2* homozygous mice, which died between 2 and 3 weeks of age (20), was not seen in *glut*2^−/−^ and MZ*glut2* zebrafish. In the present study, elevated *glut1* expression was observed in the MZ*glut2* fish intestine. Conversely, we speculated that incomplete lethality probably resulted from fish having a higher tolerance for hypoglycemia than mammals (56) or that insulin signaling has divergent roles between fish and mammals (57). Similar differences in survival between mammals and teleosts were observed in *cyp17a1* knockout fish, which would not cause lethality in fish (58), while loss of *Cyp17a1* leads to embryonic lethality in mice by embryonic day 7 (before gastrulation) (59). Therefore, in addition to compensatory *glut1* expression in the intestine, incomplete lethality could be explained by the differentiated function and requirement of GLUT2 between mammals and teleosts. Hence, zebrafish that survived after blocking glucose uptake by knocking out *glut2* had lower blood glucose levels than control fish and enhanced lipids and protein catabolism to meet energy requirements. These findings may be a potential mechanism for energy homeostasis remodeling caused by blocked glucose uptake in fish. It could also be a potential strategy for fish to adapt to hypoglycemia.

## Conclusion

In summary, we have provided new insights into the function of *glut2* and shed new light on the regulation of lipid and protein deposition by glucose uptake. The promotion of protein and amino acid degradation in muscle and lipolysis and FAO in the liver of MZ*glut2* fish are the primary adaptive metabolic responses. We suggest that *glut2*-deletion and attenuated glucose uptake cause the above phenotype decreases anabolism by inactivating insulin and increases catabolism by activating the AMPK signaling pathway. These results first demonstrated the metabolic consequences of glucose uptake attenuation in MZ*glut2* fish and provided a valuable theoretical basis and practical guidance for the establishment of a potential strategy for manipulating glucose uptake.

## Data availability statement

The original contributions presented in the study are included in the article/material, further inquiries can be directed to the corresponding authors.

## Ethics statement

All animal experiments were performed according to the Guide for Animal Care and Use Committee of Institute of Hydrobiology, Chinese Academy of Sciences (IHB, CAS, Protocol No. 2016–018).

## Author contributions

DH and ZY: conceptualization. LX, GZ, YL, YG, and QL: investigation. ZZ, HL, and JJ: methodology. LX and GZ: writing. XZ, ZY, and SX: writing, reviewing, and editing. All authors contributed to the manuscript and approved the final version.
