# Supplementary material for: Attenuated glucose uptake promotes catabolic metabolism through activated AMPK signaling and impaired insulin signaling in zebrafish
Source: Front Nutr. 2023 May 26;10:1187283. doi: 10.3389/fnut.2023.1187283 (PMC10250679; doi:10.3389/fnut.2023.1187283)
Supplement: Supplementary file 1 [file Table_1.docx]

**SUPPLEMENTARY TABLE 1** The formulation and proximate composition of the high carbohydrate diet (dry matter).

| Ingredients (g/kg) |  |
| --- | --- |
| Casein | 380.0 |
| Gelatin | 8.0 |
| Soybean oil | 50.0 |
| Dextrin | 400.0 |
| Cellulose | 100.0 |
| Mineral premix^a^ | 10.0 |
| Choline chloride | 3.0 |
| Vitamin premix^b^ | 7.0 |
| Monocalcium phosphate | 30.0 |
| L-Threonine | 1.5 |
| L-Arginine | 8.5 |
| L-Tryptophan | 2.0 |
| Proximate composition (g/kg) |  |
| Moisture | 115.6 |
| Crude protein | 372.0 |
| Crude lipid | 45.2 |
| Crude ash | 31.9 |

^a^Mineral premix (mg/kg diet): CoCO_3_, 0.65; CuSO_4_·5H_2_O, 9.00; FeSO_4_·7H_2_O, 8.34; NaCl, 400.00; MgO, 240.00; MnSO_4_·H_2_O, 22.85; KI, 0.50; Na_2_SeO_3_, 0.01; CaCO_3_,1860.00; ZnSO_4_·7H_2_O, 14.30; microcrystalline cellulose,7444.35.

^b^Vitamin premix (mg/kg diet): tocopherol acetate, 100; sodium menadione bisulfate, 25; retinyl acetate, 6.9; cholecalciferol, 0.05; thiamin, 30; riboflavin, 30; pyridoxine, 20; cyanocobalamin, 0.1; nicotinic acid, 200; folic acid, 15; ascorbic acid, 1000; inositol, 500; biotin, 3; calcium pantothenate, 100; microcrystalline cellulose 4669.95.
